# Supplementary material for: Patterns of Intron Gain and Loss in Fungi
Source: PLoS Biol. 2004 Nov 30;2(12):e422. doi: 10.1371/journal.pbio.0020422 (PMC532390; doi:10.1371/journal.pbio.0020422)
Supplement: Table S1 — Also available at http://genes.mit.edu/NielsenEtAl/. (4.3 MB ZIP). [file pbio.0020422.st001.zip › NielsenEtAl/html/1235.html]

AN1489.1.NCU00226.1.MG05309.1.FG06669.1


```
 CLUSTAL W (1.82) Multiple Sequence Alignments - Introns Inserted


Sequence 1: NCU00226.1	616 aa
Sequence 2: FG06669.1	573 aa
Sequence 3: MG05309.1	615 aa
Sequence 4: AN1489.1	533 aa
Alignment Length: 660 aa
Number Identitical Residues: 215 aa
Alignment Score (without introns) 10897


MG05309.1 	-----------MISVADIMQRLPFAASTTTRQAVTYLLGISLFSISFLVFLNSSLSFVIT
NCU00226.1	MAPTDGRPGQPFDFGAFLLRHLPFSSSTTPLQALTYLLGICLFSISFLVFLNSSVSFVIT
FG06669.1 	----------------MLLRYLPFAPSTTPLQALTYLLGISLFSISFLVFLNSSVSFVIT
AN1489.1  	----------------MASQRSMFSASR--IQVSTYLLAVCPFSIAFLVFINSSISFVVT
          	                   :   *:.*    *. ****.:. ***:****:***:***:*

MG05309.1 	DLIGVKEGVGDIVGTLGFADEIVALIACPAWGLVSDRLGVRQ~VAVVGYSIIGASLVLFV
NCU00226.1	DLIGVKHGVGDIVGTLGFVDEIVALIACPFWGLVSDRVGVRH~VAVTGYAIIGLSLLVFV
FG06669.1 	DLIGQKEGVGDVVGTLGFADELVALVACPGWGLVSDRLGVRW~VAVIGYAIIGLSLALFV
AN1489.1  	DLIGLHEGEGDAVGTLGFADELLALAACPLWGVLSDRIGVRQ0ICTAGYTIIAVALILFV
          	**** :.* ** ******.**::** *** **::***:***  :.. **:**. :* :**

MG05309.1 	QAQNVYPQLLLARILFAIGATSA2ATMVTAILPPLTDDADYSENES--DRSKKPSHNSRD
NCU00226.1	QATNVYPQLLLARILFAVGATAA2ATMVTAILPSLSDETQAQTD-----VVENPPLNPRH
FG06669.1 	QATNVYPQLLLARVLFAVGASAA2ATMVTAILPSLTDDSNSEEDEALARIKALQNATARS
AN1489.1  	QARNVYPQLLLGRLLFSIGGAAV~STMVTAILPAVTGRS------------------PRT
          	** ********.*:**::*.::. :********.::. :                  .* 

MG05309.1 	SVALSLESQVTITPERFAAQQEGGVDNSANGATAHLSDADKGDAAKPSALAGLVGLFTGC
NCU00226.1	SITPSVASEVTITPDRFRDHST-----SEAGFRFDGTQGKPEGRGKPSALAGYVGLFTGC
FG06669.1 	SIAFSVESEATITQERYTQSVS-----DRS-----MSDSADSAAGRPSKLAGFVGLFTGC
AN1489.1  	ELEEEPEPRTIVAPS--------------------------------SRLAGFVGTCAGC
          	.:  .  ... :: .                                * *** **  :**

MG05309.1 	GALVALSIFLPLPVRFGHVEGTTPAQAVTYSFYVVAAVAFCVAGFVAWGLRGIKGEEGKG
NCU00226.1	GALLALSLFLPLPARFGHIKGVTVGQAVQYSYYVVGLVSFLVAIFVFFGLRGIKGEEGKG
FG06669.1 	GALVALTLFLPLPAKFSEDKDTTPAEAVSYSFYVVAVVAMAVSLFVAIGLRNIKGEEGKG
AN1489.1  	GALISLVVLLPLPAQFQQ-WGLSPAKSIQYSYYTAAALALVVSGCCFVGLRNLPGEDGKA
          	***::* ::****.:* .  . : .::: **:*... ::: *:     ***.: **:**.

MG05309.1 	WQMLLGRSRCDDENEGDRVLSQAASLRRRRSQCRRAGQPSVQD0RYLPYGQLLKDSVRLG
NCU00226.1	FRTLFGLKRRTDD---------------------HSAPYSSK-~QVYPYLHLLRDSIRLG
FG06669.1 	FRVLFGLKDENES---------------------RSDGNGHQR0KLAPYLHLMKDSVFLG
AN1489.1  	WTSLWSPLRTEPR-----------------SFEPRKNFYSTLL~SRFPYLKQFGSAIKLG
          	:  * .                        :   :    .       ** : : .:: **

MG05309.1 	FVDPQIGLGYLGGFVARASTVAISLFIPLFINTYFIRNGFCQG-SPHDPSP-ELKKECRA
NCU00226.1	LTDSDIALGYLGGFVARASTVAISLFMPLFINAFFIRNGFCQG-SPNDPSP-ELKKECRQ
FG06669.1 	FVDSRIGLGYVGGFVARASSVAISLFIPLYVNTYYISNGFCKG-SPHDSSP-ELKEECRA
AN1489.1  	LRNRDILIGYIGGFVARASSVGISLFIPLFVNSYYHKTGLCDGQSAEGANPGDIKRSCPK
          	: :  * :**:********:*.****:**::*:::  .*:*.*.*.....*.::*..*  

MG05309.1 	AYVLASILTGVAQLFGLVCAPIFGYLASR-ATAKVKFNVPVVVATSFGIIGYILFARLQS
NCU00226.1	AYILSSILTGVAQLMGLLCAPLFGYLSSRKFTKQNRLNYPIIVATTFGIAGYIAFPQLKS
FG06669.1 	AYVLSSILTGVAQFMGLICAPVFGYLASR----TGRINYPVVVSTVFGIVGYTILPQLAS
AN1489.1  	AYVLASILTGVSQLIALIAAPAFGYLSEK----PRRYHLPLIVACLAGVVGYLLLALAPS
          	**:*:******:*::.*:.** ****:.:      : : *::*:   *: **  :.   *

MG05309.1 	PEFKDVDGRGGGPWVFLVAAMLGISQIGCIVCSLGSLGRGVLTVDLP-----------AS
NCU00226.1	PEYKDKDGRGGSPVVFFLAALIGISQIGAIVCSLGSLGRGVLKVDVVNVITHPHRVDRDS
FG06669.1 	PEIKDVEGRGGSPIIFLVVSLLGISQIGAIVCSLGSLGRGVLAVELP-------RTTRPE
AN1489.1  	PEFK---GERGNPGIFVVMALVGISQIGAIVCSLSVLSNGILSLTNS--------VEAAE
          	** *   *. *.* :*.: :::******.*****. *..*:* :               .

MG05309.1 	AQIPEEQTHASG---DERAPLLPADGAEQGGVTIRQSPAVSRVRLKGSIAGVYSWCGGAA
NCU00226.1	EVATVTEPADSR---NETAPLLENPGA-------LPEDTVSRVRLKGSVAGVYSWCGGAA
FG06669.1 	SLLQPEDYESAN---DESRPLISIT---------TDQESVSRIRLKGSIAGVYSLYGGFA
AN1489.1  	TVAGNSRAPTTGPQFDEQQPLLAAD---------RKQPQPS--DLKGSIAGIYSLFGGAG
          	          : .. :*  **:              .   *   ****:**:**  ** .

MG05309.1 	ILLLTKLGGFLFDKWSTGAPFYMMALFNAVLLVAALGVDAARF-----------YNSCK-
NCU00226.1	ILLLTKAGGYGFDEWSTGTPFYMMAGFNVVWLVVSLGIDAGRFGFKRKRGGVVIYGHQVP
FG06669.1 	ILLLTKLGGYLFDSLSNGAPFYMMAIFNAVLLAFSLGMDASRT-----------FSHQT-
AN1489.1  	ILLLTKLGGILFDVLSSDAPFYIMAGFNGVLMIACVVSGLIIT-----------LEASQ-
          	****** **  **  *..:***:** ** * :  .:  .                     

MG05309.1 	---
NCU00226.1	SGF
FG06669.1 	---
AN1489.1  	---
          	
```
